# Antenatal and postnatal combined therapy for autoantibody-related congenital atrioventricular block

**DOI:** 10.1186/1471-2393-13-220

**Published:** 2013-11-29

**Authors:** Antonio Di Mauro, Vita Caroli Casavola, Giovanna Favia Guarnieri, Grazia Calderoni, Ettore Cicinelli, Nicola Laforgia

**Affiliations:** 1 Department of Biomedical Science and Human Oncology, Neonatology and Neonatal Intensive Care Unit, University of Bari, “Aldo Moro”, P.zza Giulio Cesare, 11, 70125 Bari, Italy; 2 Department of Gynecology and Obstetrics, University of Bari “Aldo Moro”, P.zza Giulio Cesare, 11, 70125 Bari, Italy

**Keywords:** Autoantibody-associated congenital heart block, Combination therapy protocol of plasmapheresis, intravenous immunoglobulin and betamethasone in congenital heart block, Pacemaker implantation in newborn, Foetal cardiac arrhythmia detection, Foetal ecochardiography, Neonatal counselling

## Abstract

**Background:**

Autoantibody-related congenital heart block (CHB) is an autoimmune condition in which trans placental passage of maternal autoantibodies cause damage to the developing heart conduction system of the foetus.

**Case presentation:**

We report a case of an Italian 31–year-old woman, in a good clinical status, referred to our Centre at 26 weeks of her first pregnancy, because of foetal bradycardia, found during routine foetal ultrasonography. Foetal echocardiography revealed a 3rd degree CHB, without any anatomical defects. Despite the mother was asymptomatic for autoimmune disease, anti-Ro/La were searched for, because of the hypothesis of autoantibody-related CHB. High title of maternal anti-Ro/SSA antibodies was found and diagnosis of an autoantibody-related CHB was made. A combination treatment protocol of the mother was started with oral betamethasone, plasmapheresis and IVIG. An emergency C-section was performed at 32 + 3 weeks of gestation because of a non-reassuring cardiotocography pattern. A male newborn (BW 1515 g, NGA, Apgar 8–10) was treated since birth with high-flow O2 for mild RDS. IVIG administration was started at one week, and then every two weeks, until complete disappearance of maternal antibodies from blood. Because of persistent low ventricular rate (<60/min), seven days following birth, pacemaker implantation was performed. The baby is now at 40th week with no signs of cardiac failure and free of any medications.

**Conclusion:**

Up to date, no guidelines have been published for the treatment of “in utero-CHB” and only anecdotal reports are available. It has been stated that a combination therapy protocol is effective in reversing a 2nd degree CHB, but not for 3rd degree CHB. In cases of foetal bradycardia, weekly foetal echocardiographic monitoring needs to be performed and in cases of 2nd degree CHB and 3rd degree CHB maternal therapy could be suggested, as in our case, to avoid foetal heart failure. In cases of 3rd degree CHB often pacemaker implantation is needed.

## Background

Autoantibody-associated congenital heart block (CHB) is a rare neonatal disease with an overall prevalence of 1/20000 liveborn [1]. It may be detected *in utero* as a 1st- or 2nd-degree atrioventicular (AV) block, but most of the affected foetuses have a potentially lethal 3rd-degree, complete AV block [2]. Occasionally is associated a life-threatening cardiomyopathy [3].

Reported perinatal mortality rate is about 20-30% and approximately 57-66% of children born alive with CHB require pacemaker before reaching adulthood [4].

Autoantibody-associated CHB is considered a model of passively acquired autoimmune disease in which the trans-placental passage of maternal antinuclear antibodies (ANA) causes immune-mediated inflammation of the developing myocardial tissue and conduction system of the foetus [5].

Approximately 85% of foetus with congenital heart block and absence of structural abnormalities have maternal transfer of antibodies against SSA/Ro and SSB/La [6]; however only 2% of seropositive mother have newborns with congenital heart block [7]. This low risk rate rises to 19% for women with a previously affected newborn [8]. According to these data, antibodies to SSA/Ro and SSB/La could not be the only cause of the disease and other maternal and foetal factors are important [9]. Nevertheless, maternal health status is not considered a risk factor for CHB; approximately 40-60% of mothers with an affected newborn are totally asymptomatic for autoimmune disease when foetal bradycardia is found [10].

Clinical signs of conduction abnormalities (1st, 2nd, 3rd-degree heart block) most commonly develop during 18–24 weeks of pregnancy and may be found by foetal Doppler echocardiography [11]. CHB is considered a progressively developing disease and 3rd-degree heart block appears to be irreversible. Nevertheless, anecdotal cases of antenatal therapy describe the possibility of complete regression of 1st and 2nd -degree heart blocks, but only a stop of progression to heart failure for 3rd-degree heart blocks [12,13].

Up to date, no therapy has demonstrated in large case studies to be effective in preventing the progression of heart injury and in reversing autoantibody-associated CHB. We report the outcome of a combination therapy protocol described in detail in a recent paper by Ruffatti et al. [12] to treat a case of autoantibody-related 3rd-degree heart blocks referred to our Neonatal Intensive Care Unit.

## Case presentation

**Figure 1:**
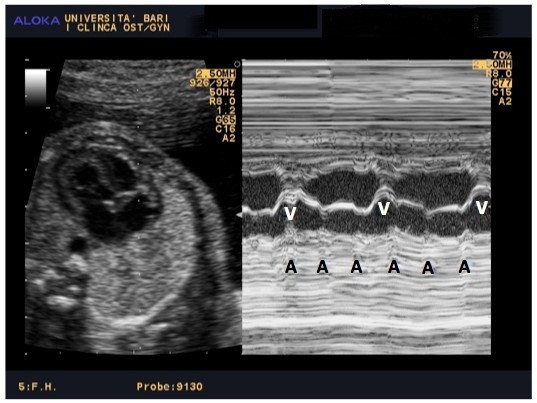
**Ultrasonograms of two-dimensional foetal echocardiograpy.** Atrial (A) and ventricular (V) contractions.

A healthy, primigravida, asymptomatic 31-year-old woman was referred to our Obstetric Unit at 26 weeks of gestation, because of the finding of foetal bradycardia during routine obstetric ultrasonography examination. The foetal echocardiography, performed in our clinic, revealed dissociation between atrial rhythm (154/bpm) and ventricular rhythm (54 bpm) (Figure 1). Neither structural heart defects nor hydrops fetalis were found.

**Figure 2:**
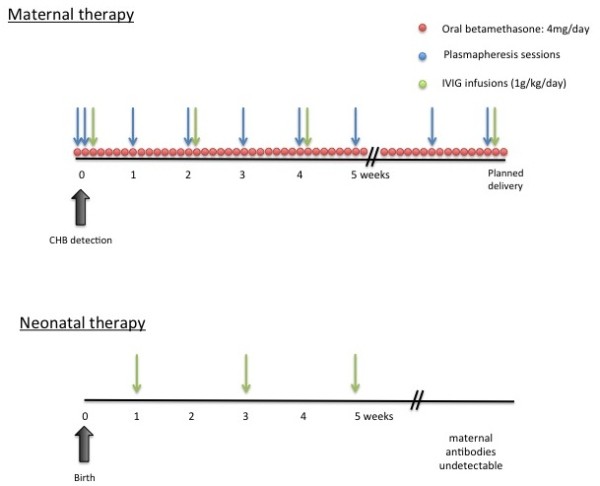
**The combination therapy protocol of plasmapheresis, intravenous immunoglobulins and betamethasone.** Oral betamethasone was daily scheduled at diagnosis until the delivery date. Plasmepheresis sessions were performed daily at onset for the first 2 days, and thereafter weekly until the delivery date. IVIG infusions was prescribed fortnightly soon after plasmapheresis. Low dose aspirin (100 mg/day) was administered to minimize the IVIG’s thrombophilia side effects.

**Figure 3:**
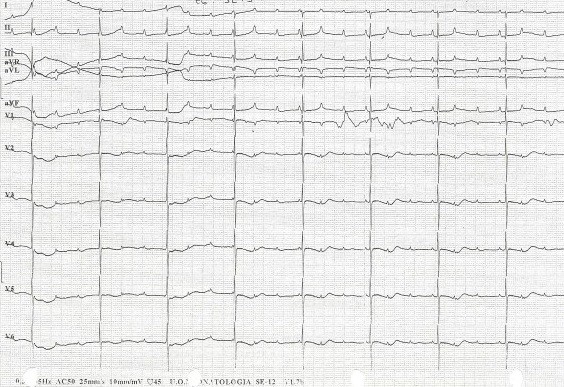
A neonatal electrocardiography shows complete atrioventricular block, with an atrial rate of 136 bpm and a ventricular rate of 44 bpm.

Despite the mother was asymptomatic for any autoimmune diseases, anti-Ro/La autoantibodies were searched for, because of the possibility of autoantibodies-related CHB. High title of maternal anti-Ro/SSA was found (359,5 U/ml) and diagnosis of an autoantibody-related CHB was made. After prenatal counselling between neonatologists, cardiologists, rheumatologists and obstetricians, mother started a combination therapy protocol of plasmapheresis, intravenous immunoglobulin and betamethasone (Figure 2). Foetal heart rate remained stable until the 32 + 3 weeks of gestation, when a non-reassuring cardiotocography pattern occurred and a 1515 g male was delivered by an emergency C- section. A male newborn (1515 g, NGA, Apgar 8–10) was treated since birth with high-flow O2 for mild RDS. Electrocardiography confirmed complete AV block, with an atrial rate of 136 bpm and a ventricular rate of 44 bpm (Figure 3), and no significant heart rate increase after *isoproterenol* (up to 0,1 μg/kg/min) administration. Newborn serum was positive for ENA (SSA/Ro = 257U/ml) and IVIG administration was started at one week and every two weeks, until complete disappearance of maternal antibodies from blood. Because of persistent low ventricular rate (<60/min), seven days following birth, pacemaker implantation was performed (Figures 3). Because of gestational age (33w + 3) and low weight (1380 g), the pacemaker (MICRONY TM II SR+; St.Jude Medical) was placed in an abdominal pocket, through a left xipho-umbilican paramedian incision, between the rectus muscle sheet and the posterior surface of the abdominal muscle, as already reported [14,15]. A 35 cm bipolar steroid-eluting epicardial pacing lead was positioned on the right ventricular free wall through a left antero-lateral thoracothomy at the 5th intercostal space. The exuberant length of the cable was rolled into the left pleural space, tunneled till the abdominal pocket and connected to the generator. The pacemaker was programmed with a heart rate of 120 bpm. The baby is now 40th week with no signs of cardiac failure and free of any medications.

## Discussion

Autoantibody-related CHB is an autoimmune condition in which trans-placental passage of maternal autoantibodies cause damage to the developing heart conduction system of the foetus. Up to date, no therapy is found to be effective in the treatment of 2nd and 3rd degree CHB. Ruffatti et al. have recently showed the possibility of a combination strategy in order to revert or block the progression of immune-related heart damage [12,13]. The rationale behind this combined protocol is that it is possible to obtain a sum of the effects of each drug: a) bethametasone, a fluorinated steroids only partially inactivated by the placenta, reduces inflammation of the developing conduction system; b) plasmapheresis lowers the level of the offending antibodies; c) IVIG made an anti-idiotype regulation, inhibiting the placental transport of maternal antibodies, and modulating macrophages response with secondary reduction of inflammation and fibrosis of the foetal heart.

This is a report of an antibody-related complete CHB, treated in utero with a combination therapy protocol, a pacemaker implantation at one week of age and bi-weekly IVIG administration to the newborn.

According to the protocol, the newborn was also treated with IVIG after birth, and every two weeks, until maternal autoantibodies became undetectable. The treatment continued in post-natal period because, although the pacemaker implantation preserve electrophysiological heart failure, it does not avoid myocardial injury from persistent maternal autoantibodies that may clinically manifests with myocardial dysfunction after birth, even with adequate pacemaker therapy [16]. We have also avoided breastfeeding, because of the possible transfer human of anti-Ro/La antibodies via breast milk and subsequent tissue injury [17]. This post-natal treatment may accelerate the clearance of potentially pathogenic autoantibodies and avoid the risk of myocardial injury (fibrosis) progression.

No side effects related to the protocol were observed both in the mother and newborn. Despite the case reported by Ruffatti et al. [12] showed an improvement of foetal heart rate, we were not able to demonstrate this effect in our foetus with 3rd degree AV block, and an early pacemaker implantation was needed after birth, at one week of age. Probably, the timing of starting the combined protocol during pregnancy might affect the result in term of foetal heart rate improvement. Further studies are needed to demonstrate the correct timing and efficacy of this combined protocol.

## Conclusion

Different therapeutic regimens have been proposed for foetal/neonatal CHB, but controlled clinical trials are not yet available. CHB is a progressive disease and, presumably, the best time for any therapy is during early stages of pregnancy, when inflammation of the heart conduction system, but not fibrosis, is present. Thus, early assessment of the foetus and serial foetal echocardiographs to identify reversible block are needed, to start treatment when CHB is still incomplete [18]. Multidisciplinary prenatal counseling is fundamental for the best treatment, both for foetus and newborn.

## Consent

Written informed consent was obtained from the patient for publication of this case report and any accompanying images. A copy of the written consent is available for review by the Editor of this journal.

## Abbreviations

CHB: Congenital heart block; AV: Atrioventricular; Bpm: Beat per minute; ANA: Antinuclear antibodies.

## Competing interest

The authors declare that they have no competing interest.

## Authors’ contributions

GC and VCC participated in the literature search and in selected relevant papers. GFG resolved inconsistencies with discussion. NL and EC was involved in revising the final draft of the manuscript. ADM was responsible for the concept, design and writing of the final version of the manuscript. All authors read and approved the final manuscript.

## Pre-publication history

The pre-publication history for this paper can be accessed here:

http://www.biomedcentral.com/1471-2393/13/220/prepub
